# Inhibitory Effects of 6,8-Diprenylorobol on Endometriosis Progression in Humans by Disrupting Calcium Homeostasis and Mitochondrial Function

**DOI:** 10.3390/antiox11010171

**Published:** 2022-01-17

**Authors:** Jisoo Song, Gwonhwa Song, Sunwoo Park, Whasun Lim

**Affiliations:** 1 Department of Biological Sciences, Sungkyunkwan University, Suwon 16419, Korea; js_song97@kookmin.ac.kr; 2 Department of Food and Nutrition, College of Science and Technology, Kookmin University, Seoul 02707, Korea; 3 Institute of Animal Molecular Biotechnology, Department of Biotechnology, College of Life Sciences and Biotechnology, Korea University, Seoul 02841, Korea; ghsong@korea.ac.kr; 4 Department of Plant & Biomaterials Science, Gyeongsang National University, Jinju-si 52725, Korea

**Keywords:** 6,8-diprenylorobol, endometriosis, calcium homeostasis, proliferation, ROS

## Abstract

6,8-Diprenylorobol is a flavonoid compound extracted from *Cudrania tricuspidata*. It has various biological functions, such as inhibiting melanin synthesis and inducting cell death in cancerous cells. In addition, *Cudrania tricuspidata* is known to be effective in female diseases, and previous studies have shown anticancer effects in cervical cancer, a female reproductive disease. Outside of that, *Cudrania tricuspidata* has various physiological effects. However, the effect of 6,8-diprenylorobol is not well known in other benign and chronic diseases, even in endometriosis, which commonly arises in the female reproductive tract. In the present study, we determined the inhibitory effects of 6,8-diprenylorobol on the growth of endometriosis VK2/E6E7 and End1/E6E7 cells. Results indicated that 6,8-diprenylorobol suppressed cellular proliferation and increased the disruption of the cell cycle, mitochondrial membrane potential (MMP), generation of reactive oxygen species, and Ca^2+^ homeostasis in both endometriosis cells. However, the proliferation of normal stromal cells isolated from endometrial tissue was not altered by 6,8-diprenylorobol. The change in Ca^2+^ levels was estimated in fluo-4- or rhod-2-stained VK2/E6E7 and End1/E6E7 cells after the treatment of the intracellular calcium regulators 2-aminoethoxydiphenyl borate (2-APB) and ruthenium red (RUR) with 6,8-diprenylorobol. A combination of 6,8-diprenylorobol with each regulator decreased the calcium accumulation in endometriosis cells. Furthermore, Western blot analysis indicated that 6,8-diprenylorobol inactivated AKT pathways, whereas it activated P38 MAPK pathways. In addition, 6,8-diprenylorobol decreased mitochondrial respiration, leading to the reduction in ATP production in VK2/E6E7 and End1/E6E7 cells. Collectively, our results suggested that 6,8-diprenylorobol might be a potential therapeutic agent or adjuvant therapy for the management of endometriosis.

## 1. Introduction

Endometriosis (EMS) is a gynecological disease, defined as the abnormal presence of endometrial-like tissue, such as ovarian, pelvic, intestinal, or fallopian tubes, outside of a woman’s womb. The age for prevalence of endometriosis is 25–35 years, and it increases the prevalence of ovarian cancer to more than 50% compared to a healthy woman [1]. Further, an estrogen-dependent chronic inflammation process is a hallmark of endometriosis, and the symptoms of EMS include pelvic pain, menstrual pain, pain with bowel movements, and infertility [2]. The cause of endometriosis is not clearly known, but the pathologic retrograde menstruation theory, which explains that backward menstrual blood flow cannot be removed in the abdominal cavity, is usually accepted. This theory is reported to be related to immunology [3]. The major therapeutic methodologies of endometriosis are surgery or hormone therapy; however, the surgery for endometriosis should be repeated, and hormone therapy drugs, including GnRH-a and progestin, have side effects such as bone loss, amenorrhea, weight gain, and depression [4]. Therefore, with unclear causes of the development of endometriosis, the patients’ lives are negatively impacted simultaneously. Hence, as a consequence, the treatment method for endometriosis should be enhanced. 

Phytochemicals belong to the second metabolite of plants and various chemicals such as polyphenols, flavonoids, coumarin, and steroid saponin. These phytochemicals have been previously used in conventional medicine therapies [5]. Flavonoids are one of the phytochemical classes, and have several health-enhancing effects, such as antioxidation, anti-cell proliferation, anticancer, and anti-inflammation [6]. *Cudrania tricuspidata* has been used as a folk remedy for a long time, and is regarded as good for insomnia, weakness, and female diseases [7]. In addition, various physiological effects of *Cudrania tricuspidata* have been determined through previous studies. Representatively, there was a study to inhibit cell proliferation by increasing the expression of p53 and inducing apoptosis through the extracellular pathway when the *Cudrania tricuspidata* stem extract was used to treat cervical cancer [8]. In addition, the study showed anticancer effects in breast and colorectal cancer [9,10]. Another study showed that it has neuroprotective and cytoprotective effects [11,12]. Through this, a study was conducted to find out the effect of 6,8-diprenylorobol, which is one of the *Cudrania tricuspidata* extracts showing various effects. 6,8-Diprenylorobol belongs to the class of flavonoids and can be extracted from the leaves of *Cudrania tricuspidata* [13]. 6,8-Diprenylorobol exhibits anti-cell proliferation, anticancer, antifungal and anti-Helicobacter pylori activities [14,15]. For example, 6,8-diprenylorobol indicated an anticancer effect that induced apoptosis in hepatocellular carcinoma cell lines by inhibiting cytochrome P450 family 2 subfamily J member 2 (CYP2J2) and activating forkhead box O3 (FOXO3) [16]. In addition, 6,8-diprenylorobol induced apoptosis in colon cancer by activating the P53 apoptotic cell signal pathway [17]. However, the antiproliferation effects of 6,8-diprenylorobol on human endometriosis have not yet been identified. 

Thus, we examined the effects of 6,8-diprenylorobol on endometriosis for the following: (1) suppression of cellular proliferation; (2) induction of cell cycle arrest; (3) impairment of mitochondrial function and calcium homeostasis; (4) dysregulation of the intracellular signaling pathway (PI3K/AKT signal); and (5) changes in PI3K/AKT protein expression by 6,8-diprenylorobol with inhibitor. 

## 2. Materials and Methods

### 2.1. Reagents

The 6,8-diprenylorobol (Cat. No. CFN97705) was purchased from Chem Faces (Wuhan, China) and dissolved in dimethyl sulfoxide (Sigma-Aldrich, St. Louis, MO, USA). The antibodies against phosphorylated AKT (Ser^473^, Cat. No. 4060), P70S6K (Thr^421^/Ser^424^, Cat. No. 9204), S6 (Ser^235/236^, Cat. No. 2211), and p38 (Thr^180^/Tyr^182^, Cat. No.4511); and the total form of AKT (Cat. No. 9272), P70S6K (Cat. No. 2708), S6 (Cat. No. 2217), and P38 (Cat. No.9212) were purchased from Cell Signaling Technology (Beverly, MA, USA). The 2-APB (Cat. No. D9754) was purchased from Sigma-Aldrich. The ruthenium red (Cat. No. Ab120264) was purchased from Abcam (Cambridge, UK). The inhibitor of PI3K/AKT (LY294002, Cat. No. 9910) was also purchased from Cell Signaling Technology.

### 2.2. Cell Culture Method

The human endometriosis-like cell lines VK2/E6E7 and End1/E6E7 were purchased from the American Type Culture Collection (Manassas, VA, USA). As the cell culture medium, keratinocyte serum-free medium (Cat. No. 17005-042, Gibco, Waltham, MA, USA) and DMEM/F12 1:1 medium (Cat. No. SH30023.01, Cytiva, Marlborough, MA, USA) containing 10% fetal bovine serum (FBS) were used. The primary normal endometrial epithelial cells were purchased from the Lifeline Cell Technology (Frederick, MD, USA). The normal epithelial cells were cultured in ReproLife^TM^ reproductive medium (Cat. No. LL-0068, Lifeline Cell Technology) according to the manufacturer’s guidelines. The cells were incubated in 100 mm cell culture dishes until 70% confluence, and treated with different concentrations of 6,8-diprenylorobol with or without a calcium inhibitor for 48 h.

### 2.3. Cell Proliferation Measurements

The proliferation assay was performed using the BrdU ELISA kit (Roche, Basel, Switzerland) according to the manufacturer’s instructions and as described in a previous study [12]. Concisely, cells were cultured in a 96-well plate, and endometriosis cells were incubated with dose-dependent 6,8-diprenylorobol in a maximum volume of 100 μL/well for 48 h. After BrdU labeling, the cells were fixed, and anti-BrdU-POD was added for 90 min. The absorbance was detected as wavelengths at 370 nm and 420 nm by microplate spectrophotometer, and each treatment was performed three times

### 2.4. Immunofluorescence Detection of PCNA 

The effect of 6,8-diprenylorobol on the expression level of proliferative cell nuclear antigen (PCNA) was determined by immunofluorescence microscopy. Concisely, cells (5 × 10^3^ cells) were seeded on confocal dishes. Then, the cells were incubated with 6,8-diprenylorobol (2 μM) for 48 h at 37 °C in a 5% CO_2_ incubator. After treatment, the cells were washed and blocked with goat serum and stained with a primary PCNA (Cat. No. sc-56, Santa Cruz Biotechnology). Then, a secondary antibody for PCNA (Cat. No. A-11001, Invitrogen, Carlsbad, CA, USA) and 4′,6-diamidino-2-phenylinodole (DAPI, Cat. No. D8417, Sigma) was added. The fluorescence of the confocal dish was captured by confocal microscope (LSM710, Carl Zeiss). The fluorescence was measured using three different images of each sample and compared to the vehicle-treated cell, which was indicated the bar graph. More detailed processes of this assay were described in the previous study [18].

### 2.5. Cell Cycle Progression Analysis

The changes in the cell cycle stage of VK2/E6E7 and End1/E6E7 by 6,8-diprenylorobol were detected using propidium iodide (PI; BD Biosciences, Franklin Lakes, NJ, USA). Concisely, both types of cells (2 × 10^5^ cells) were seeded in 6-well plates and treated with 6,8-diprenylorobol (0, 0.1, 0.2, 0.5, 1, and 2 μM) for 48 h at 37 °C in a 5% CO_2_ incubator. Subsequently, the cells were fixed in 0.1% BSA phosphate-buffered saline (PBS) and chilled in 70% ethanol at 4 °C for 16 h. The cells were treated with 10 mg/mL RNase A (Sigma-Aldrich) and 50 mg/mL PI, and then incubated for 30 min at 25 °C. The results were measured at 1 × 10^4^ cells with a BD FACSCalibur, and each assay was independently performed in triplicate. This assay was performed in accordance with a previous study [18].

### 2.6. JC-1 MMP Assay

Changes in the MMP of VK2/E6E7 and End1/E6E7 cells were analyzed using a mitochondrial staining kit (Cat. No. CS0390, Sigma-Aldrich). According to the manufacturer’s manual, prepared endometriosis cells were stained with JC-1 staining solution and incubated for 20 min at 37 °C in CO_2_ incubators. After washing with staining buffer, JC-1-stained cells (1 × 10^4^ cells) were detected using a FACSCalibur. The results compared to vehicle-treated cells were indicated in a bar graph. Each assay was performed three times independently. This assay was performed in accordance with a previous study [18].

### 2.7. ROS Assay

The increased level of intracellular reactive oxygen species (ROS) production by 6,8-diprenylorobol treatment was detected by using 2′-7′-dichlorofluorescein diacetate (DCFH-DA, Sigma-Aldrich), which was converted to 2′-7′-dichlorofluorescin (DCF) by peroxides. Concisely, both types of cells were treated with 10 μM of DCFH-DH and then washed with 1× PBS. The cells (1 × 10^4^ cells) were measured using a FACSCalibur, and the experiment was performed independently three times. This assay was performed in accordance with a previous study [18].

### 2.8. Determination of Intracellular Calcium Ion Concentration Assay

The calcium ion level in the cytosol was analyzed using fluo-4 AM dye (Invitrogen). Concisely, 6,8-diprenylorobol-treated cells were stained with 3 μM fluo-4 AM for 20 min, and the stained cells were washed with 1× PBS. In addition, the cells (1 × 10^4^ cells) were detected using a FACSCalibur, and the results compared to vehicle-treated cells were indicated in a bar graph. Each assay was independently performed three times. This assay was performed pursuant to a previous study [18].

### 2.9. Determination of Mitochondrial Matrix Calcium Ion Concentration Assay

The calcium ion concentration levels in the mitochondria were detected using 3 μM rhod-2 AM (Invitrogen). Concisely, identical cell preparation was as described above, and collected cells were stained with rhod-2 AM for 30 min. Further, Hank’s balanced salt solution (HBSS, Gibco) was dispensed into the stained cells and incubated for 10 min. Then, the 1 × 10^4^ cells were measured by FACS, and the ratio of calcium accumulation was indicated in a bar graph. This assay was performed pursuant to a previous study [18]. 

### 2.10. Determination of Mitochondrial Respiration

We detected mitochondrial respiration using a Seahorse XFe 24 analyzer (Agilent Technologies, Santa Clara, CA, USA). An XF Cell Mito Stress kit was purchased from Agilent Technologies for conducting experiments according to the manufacturer’s instruction. The VK2/E6E7 and End1/E6E7 cells were seeded in a 24-well cell culture microplate at a concentration of 6 × 10^4^ cells/well. Cells were treated with 6,8-diprenylorobol (2 μM) in keratinocyte culture medium at 37 °C in a CO_2_ incubator for 16 h. Then, the VK2/E6E7 and End1/E6E7 cells were serially treated with oligomycin (1.5 μM), FCCP (0.5 μM), rotenone, and antimycin A (0.5 μM) to calculate various mitochondrial respiration parameters. Data for cell lines were detected three times.

### 2.11. Western Blot Assay

The protein was extracted from each whole cell and the concentration determined via a Bradford assay (Bio-Red, Hercules, CA, USA) with BSA as the standard. The proteins were denatured and isolated via 10% sodium dodecyl sulfate–polyacrylamide gel electrophoresis (SDS-PAGE) and then transferred to a nitrocellulose membrane. Primary and secondary antibodies for each protein were dispensed in serial order and measured using a ChemiDoc EQ system and Quantity One software (Bio-Rad). The results were measured using three cell culture plates, and changes in phosphorylation were indicated in a bar graph. The assay was performed as described in a previous study [18].

### 2.12. Statistical Analysis

All data were subjected to analysis of variance pursuant to the general linear model (PROC-GLM) of the SAS program (SAS Institute, Cary, NC, USA) to confirm whether there were significant differential effects on each cell type in response to treatments. Differences with a probability value of *p* < 0.05 were considered to be statistically significant. Data are presented as mean ± standard error of the mean unless otherwise stated.

## 3. Results

### 3.1. 6,8-Diprenylorobol Suppresses Proliferation of Human Endometriosis-like Cell Lines

**Figure 1:**
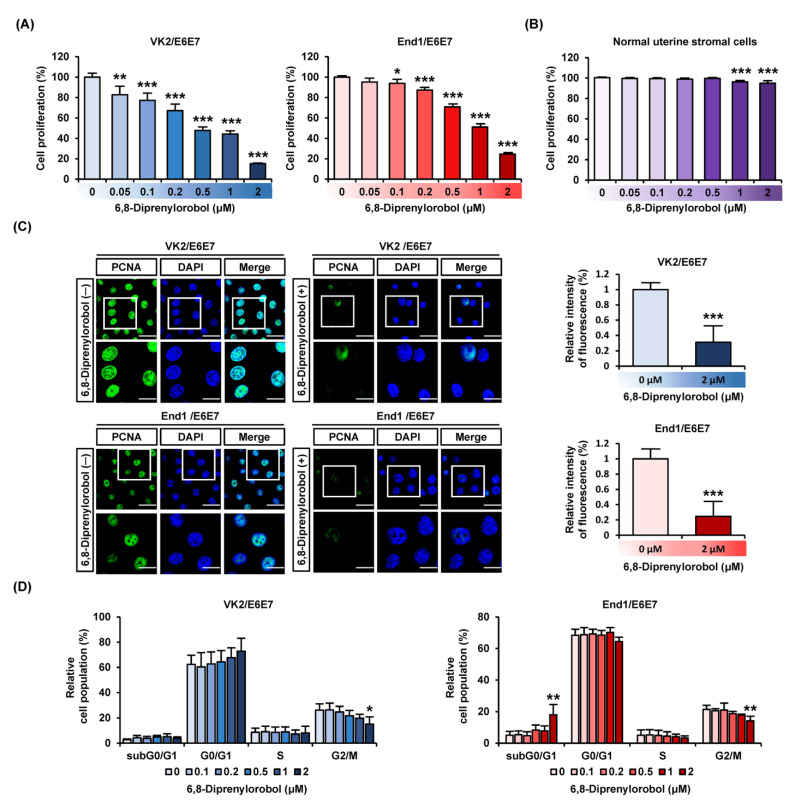
Antiproliferation effects of 6,8-diprenylorobol in human endometriosis cells. (**A**) Cell proliferation of VK2/E6E7 and End1/E6E7 in response to various concentrations of 6,8-diprenylorobol (0, 0.1, 0.2, 0.5, 1, and 2 μM) was conducted. Average values of triplicated data were converted to relative ratio values and represented in a bar graph. (**B**) Proliferation of normal uterine stromal cells was treated with 6,8-diprenylorobol. (**C**) Confocal images of VK2/E6E7 and End1/E6E7cells were captured. Green fluorescence indicated PCNA, and blue fluorescence indicated DAPI. The relative intensity of fluorescence between the vehicle and 6,8-diprenylorobol (2 μM) treatment was represented as a bar graph. (**D**) Cell cycle arrest of VK2/E6E7 and End1/E6E7 cells was affirmed by propidium iodide (PI) by FACS. Asterisks indicate significant levels between vehicle-treated cells and 6,8-diprenylorobol-treated cells (* *p* < 0.05, ** *p* < 0.01, and *** *p* < 0.001).

We detected cell proliferation in various concentrations of 6,8-diprenylorobol (0, 0.05, 0.1, 0.2, 0.5, 1, and 2 μM) in VK2/E6E7 and End1/E6E7 cells, as illustrated in Figure 1A. Our results indicated that 2 μM of 6,8-diprenylorobol reduced proliferation by more than 50% in both cell types. In addition, to confirm the toxicity of 6′,8-diprenylorobol in normal endometrial cells, the cell proliferation was performed using primary human normal uterine stromal cells. Accordingly, only 8% of cell proliferation was decreased by 6,8-diprenylorobol, compared to vehicle-treated cells (Figure 1B). Furthermore, when VK2/E6E7 and End1/E6E7 cells were treated with 6,8-diprenylorobol, the relative expression of green fluorescence (PCNA), representing a proliferation marker, was reduced by more than 50% compared to vehicle-treated cells (Figure 1C). Moreover, we utilized propidium iodide (PI) staining to confirm that 6,8-diprenylorobol caused cell cycle arrest in both cell types (Figure 1D). In response to 6,8-diprenylorobol treatment with varying concentrations (0, 0.1, 0.2, 0.5, 1, and 2 μM), the relative cell population of the subG0/G1 phase in End1/E6E7 cells was gradually increased. In addition, the G2/M phase cell population in both cell types was gradually decreased by 6,8-diprenylorobol treatment (Figure 1D). These results revealed that 6,8-diprenylorobol reduced the proliferation of human endometriosis-like cell lines.

### 3.2. 6,8-Diprenylorobol Induces Loss of MMP and Increases ROS Production in Human Endometriosis-like Cell Lines

**Figure 2:**
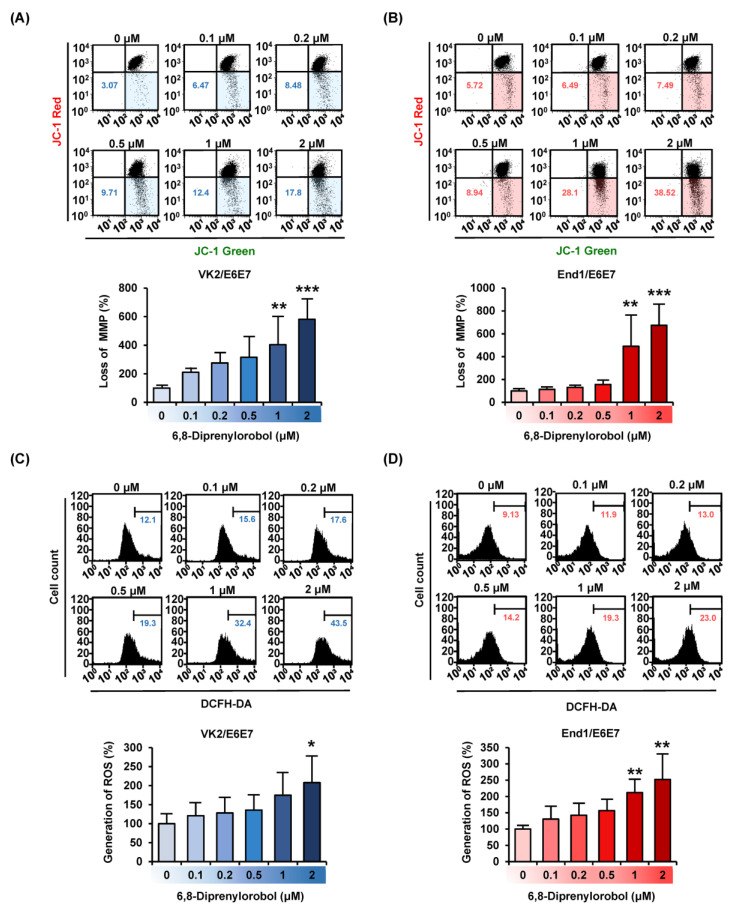
Effects of 6,8-diprenylorobol on mitochondrial function and ROS production in VK2/E6E7 and End1/E6E7 cells. (**A**,**B**) MMP in VK2/E6E7 and End1/E6E7 cells was confirmed by JC-1 dye with flow cytometry (FACS). (**C**,**D**) The human endometriosis cells were stained with DCFH-DA to detect the production of reactive oxygen species (ROS) via FACS. Asterisks indicate significant levels between vehicle-treated cells and 6,8-diprenylorobol-treated cells (* *p* < 0.05, ** *p* < 0.01, and *** *p* < 0.001).

We investigated the effects of 6,8-diprenylorobol on mitochondrial function in human endometriosis cells by measuring MMP (∆ψ) and generating ROS. Our results revealed that 6,8-diprenylorobol induced the depolarization of the mitochondrial membrane in both cell lines (Figure 2A,B). The 2 μM of 6,8-diprenylorobol in both cells significantly raised the relative MMP loss ratio up to 581% (*p* < 0.001) in VK2/E6E7 and 673% (*p* < 0.001) in End1/E6E7. In addition, we examined the production of ROS in response to the 6,8-diprenylorobol treatment. The relative percentage of ROS production was increased by up to 207% (*p* < 0.05) in VK2/E6E7 and 252% (*p* < 0.01) in End1/E6E7 treated with 2 μM of 6,8-diprenylorobol compared to vehicle-treated cells (Figure 2C,D). Based on these results, we demonstrated that 6,8-diprenylorobol induced mitochondrial dysfunction and inhibited the oxidative stress buffering system.

### 3.3. 6,8-Diprenylorobol Disrupts Calcium Homeostasis in Cytosol and the Mitochondrial Matrix in Human Endometriosis-like Cell Lines 

**Figure 3:**
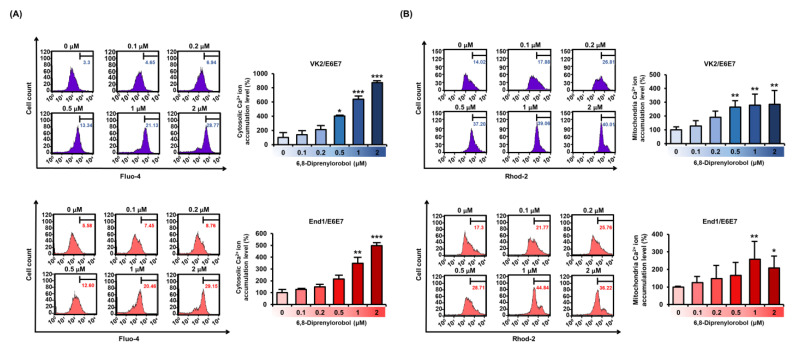
Effects of 6,8-diprenylorobol on disrupting calcium homeostasis in VK2/E6E7 and End1/E6E7. (**A**) FACS analysis of cytosolic calcium ion accumulation in VK2/E6E7 and End1/E6E7 cells stained with fluo-4 AM dye. (**B**) The accumulation of mitochondrial calcium in VK2/E6E7 and End1/E6E7 cells was measured by staining with rhod-2 AM. The right part of the histogram’s peak was measured, and its values were converted to a bar graph based on a percentage-ratio. Asterisks indicate significant levels between vehicle-treated cells and 6,8-diprenylorobol-treated cells (* *p* < 0.05, ** *p* < 0.01, and *** *p* < 0.001).

**Figure 4:**
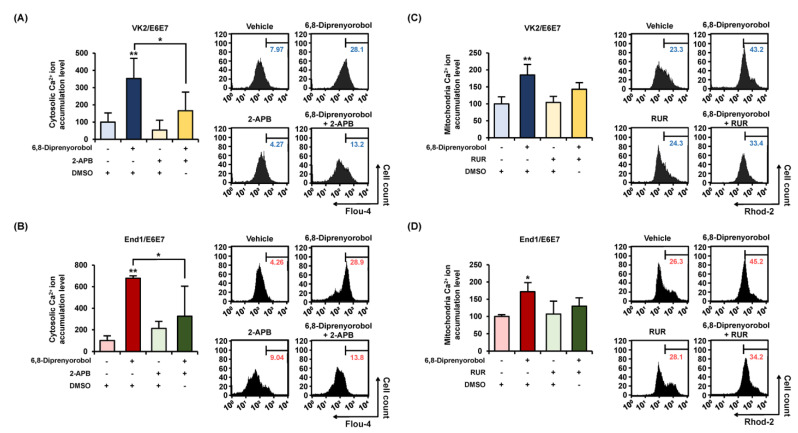
Alleviation effects of 6,8-diprenylorobol on disrupting calcium homeostasis in VK2/E6E7 and End1/E6E7. For 48 h, 6,8-diprenylorobol (2 μM) was treated with or without a calcium inhibitor (2-APB, 5 μM; and ruthenium red, 8 μM). (**A**,**B**) FACS was adopted to assess the alleviation effects of cytosolic calcium accumulation in human endometriosis cells. (**C**,**D**) Alleviation effects of the accumulation of mitochondrial matrix calcium was confirmed in human endometriosis cells by FACS. Asterisks indicate significant levels between vehicle-treated cells and 6,8-diprenylorobol-treated cells (* *p* < 0.05 and ** *p* < 0.01).

Calcium homeostasis disruption could lead to mitochondrial dysfunction. Therefore, to measure the interfering effect of 6,8-diprenylorobol on calcium homeostasis in human endometriosis-like cells, we conducted fluo-4 and rhod-2 dye staining of both cell lines. An increase in fluo-4 and rhod-2 dyes represented the calcium accumulation in the cytosol and mitochondrial matrix, respectively. Intracellular cytosolic calcium levels were gradually upregulated by 6,8-diprenylorobol, up to 827% in VK2/E6E7 and 498% in End/E6E7 compared to vehicle-treated cells (Figure 3A). In addition, mitochondrial calcium levels of 6,8-diprenylorobol-treated cells were increased by 285% and 258% in VK2/E6E7 and End1/E6E7 cells, respectively, compared to vehicle-treated cells (Figure 3B) Furthermore, we executed the changes in cytosolic calcium and mitochondrial matrix calcium with 2-aminoethoxydiphenyl borate (2-APB) or ruthenium red (RUR), which are calcium inhibitors, to verify the mechanism of calcium influx by 6,8-diprenylorobol in the human endometriosis-like cell lines. Relative calcium accumulation in cytosol was mitigated from 352% to 165% in VK2/E6E7 cells, and from 678% to 325% in End1/E6E7 by cotreatment of 2-APB and 6,8-diprenylorobol, compared to cells solely treated with 6,8-diprenylorobol (Figure 4A,B). Similarly, the accumulation of calcium ions in the mitochondrial matrix was mitigated from 185% to 142% in VK2/E6E7, and from 171% to 130% in End1/E6E7 by cotreatment of ruthenium red and 6,8-diprenylorobol, compared to 6,8-diprenylorobol solo treatment. However, there was no significant difference in RUR cotreatment as compared to 6,8-diprenylorobol alone. It seemed that accumulation of calcium ions by 6,8-diprenylorobol was primarily in the cytosol.

### 3.4. 6,8-Diprenylorobol Regulates Mitochondrial Respiration in Human Endometriosis-like Cell Lines

**Figure 5:**
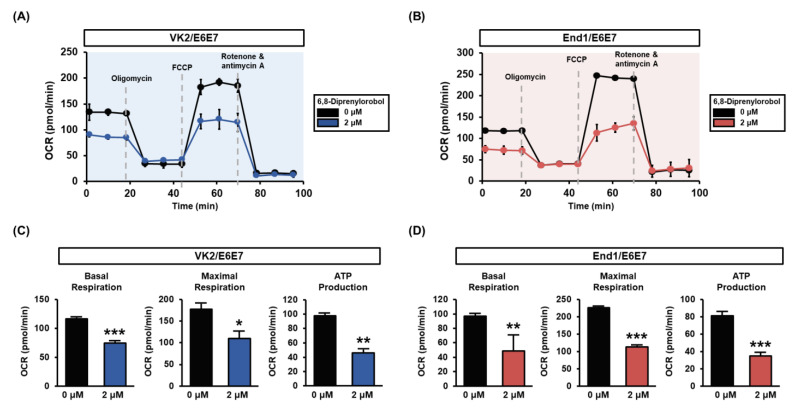
Effects of 6,8-diprenylorobol on mitochondrial respiration of VK2/E6E7 and End1/E6E7 cells. (**A**,**B**) Mitochondrial respiration was measured in VK2/E6E7 and End1/E6E7 with a Seahorse XFe analyzer. (**C**,**D**) Each part of basal respiration, maximal respiration, and ATP production between the vehicle and 6,8-diprenylorobol groups is indicated as a graph. Asterisks indicate significant levels between vehicle-treated cells and 6,8-diprenylorobol-treated cells (* *p* < 0.05, ** *p* < 0.01, and *** *p* < 0.001).

Mitochondrial respiration of VK2/E6E7 and End1/E6E7 cells was measured with a Seahorse XFe analyzer. The mitochondrial respiratory rates of each phase are illustrated in Figure 5. The basal respiratory rates of 6,8-diprenylorobol-treated cells, prior to oligomycin treatment, were lower than those of vehicle-treated cells. Similarly, after the FCCP induction, the maximal respiratory rates of 6,8-diprenylorobol-treated cells were lower than those of the vehicle-treated cells in both cell lines. Moreover, the ATP-linked respiration was decreased by 6,8-diprenylorobol treatment in both cell lines. Using these results, we clearly confirmed that 6,8-diprenylorobol influenced the mitochondrial dysfunction.

### 3.5. 6,8-Diprenylorobol Downregulates the Phosphorylation of the Intracellular Signaling Pathway in Human Endometriosis-like Cell Lines 

**Figure 6:**
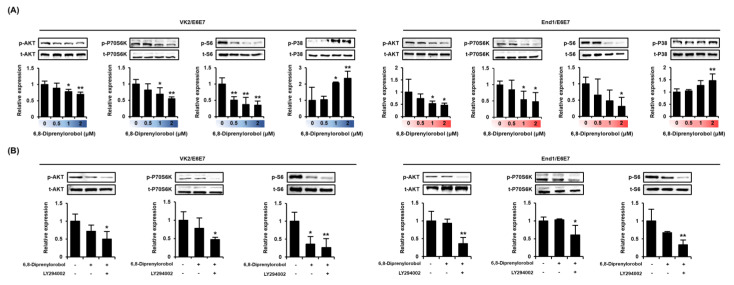
Regulation of PI3K/AKT signaling pathways by 6,8-diprenylorobol in human endometriosis cells. (**A**) Phosphorylation of AKT, P70S6K, S6, and P38 in response to dose-dependent treatment of 6,8-diprenylorobol. (**B**) Phosphorylation of AKT, P70S6K, and S6 in response to LY294002 treatment with 6,8-diprenylorobol. Immunoblots were captured and digitized using a ChemiDoc EQ system and Quantity One software. Each value was normalized by each total form of proteins and represented as fold changes in the graph. Asterisks indicate significant levels between vehicle-treated cells and 6,8-diprenylorobol-treated cells (* *p* < 0.05 and ** *p* < 0.01).

We measured the phosphorylation status of PI3K/AKT using signaling proteins and P38 proteins in 6,8-diprenylorobol, and treated VK2/E6E7 and End1/E6E7 cells for 24 h (Figure 6A). The phosphorylation of AKT, P70S6K, and S6 decreased as the dose of 6′,8-diprenylorobol increased. However, the phosphorylation of P38 protein was increased in accordance with the 6,8-diprenylorobol treatment. Further, the inhibitory effects of pharmacological inhibitor LY294002 in the presence of 2 μM of 6,8-diprenylorobol were analyzed (Figure 6B). We confirmed that the phosphorylation of PI3K/AKT proteins was effectively inhibited by cotreatment with 6,8-diprenylorobol and LY294002 in both cell lines, compared to cells solely treated with 6,8-diprenylorobol.

## 4. Discussion

Until recently, 6,8-diprenylorobol, one of the flavonoids, had not been studied actively. Even these limited studies of the bioactive effects of 6,8-diprenylorobol have mainly been conducted for cancers. In a previous study, 6,8-diprenylorobol exhibited antiproliferative and apoptotic effects, and increased the production of ROS in colon cancer [17]. In addition, 6,8-diprenylorobol suppressed cell viability and induced apoptosis in human liver cancer cells (HCC) via regulation of FOXO3 and CYP2J2 [16]. In addition, a previous study suggested that 6,8-diprenylorobol acted as an inhibitor of aromatase in breast cancer [19]. Therefore, we examined its potent therapeutic effects in human endometriosis. Similar to previous studies, in this study, 6,8-diprenylorobol affected cell survival in human endometriosis-like cells, with various changes in the intracellular organelles and levels of signaling proteins. 

Calcium (Ca^2+^) signaling regulates various physiological processes; the intracellular calcium ion level is critical for cell survival, cell function, and mitochondrial dynamics. It is known that intracellular calcium regulates various cellular functions, such as mitochondrial metabolism and cell proliferation [20]. Ca^2+^ mainly forms a complex to regulate cell proliferation and death, and Ca^2+^ regulates the cell cycle through the G1 checkpoint, G2/M, and spindle assembly checkpoints [21]. Any change in the process can lead to cell cycle arrest and death [22]. Increased cytosolic calcium stimulates calcium-sensitive proteins to propagate signals, and affects cell survival as well. In the present study, 6,8-diprenylorobol induced antiproliferative effects with cell cycle arrest in VK2/E6E7 and End1/E6E7 cells. In addition, calcium could stimulate transcription through the cAMP response element binding protein (CREB) in the presence of CaM II and CaMK IV in the nucleus [23,24]. Therefore, calcium was excessively accumulated when losing the MMP, and finally, apoptosis was induced [25,26]. Similarly, 6,8-diprenylorobol induced the depolarization of mitochondrial membranes and calcium overload in the cytosol and mitochondrial matrix in this study. To verify the specific mechanism of 6,8-diprenylorobol in calcium homeostasis, we utilized two types of calcium inhibitors, 2-APB and RUR. The 2-APB inhibited the IP3 receptor through membrane penetration of the calcium storage other than mitochondria [27,28,29]. Ruthenium red is an inhibitor of the mitochondrial matrix calcium uniporter, and it inhibits calcium uptake into the mitochondrial matrix [30,31]. In the present study, we confirmed that the excessive calcium accumulation by 6,8-diprenylorobol was diminished with 2-APB treatment. Therefore, we found that 6,8-diprenylorobol influenced calcium regulation through IP3 receptors in human endometriosis-like cells. 

Mitochondria play an important role in various cell functions with energy production. They produce cellular energy through oxidative oxidation (OXPHOS), and the OXPHOS complex comprises mitochondrial complexes I–V. The maximal capacity of cellular oxidative phosphorylation is an important determinant of cell survival [32], and functional impairment of mitochondrial complex I has been associated with various human diseases. Recently, a few mitochondrial DNA (mtDNA) mutations in complex I subunit encoding genes were observed in endometriosis patients. These mutations affect the normal electron transport chains and increase ROS production, which is one of the causes of endometriosis [33]. These results suggested that cellular respiration by mitochondria plays an important role during the pathogenesis and development of endometriosis. Currently, it has been reported that several drugs acting on the mitochondrial electron transport chain exhibited anticancer effects [34,35]. While few such studies have been conducted on endometriosis, we confirmed that mitochondrial dysfunction was related to mitochondrial respiration and metabolism through this study. Therefore, we speculated that mitochondrial respiration will affect the treatment mechanism of endometriosis, based on the results of previous studies and this study. Therefore, this study confirmed that 6,8-diprenylorobol affected cellular energy production with lower mitochondrial respiration. 

PI3K is a known major regulator for cell survival and apoptosis [36]. Therefore, downregulation of the PI3K/AKT/mTOR pathway is generally suggested as a therapeutic target for cancer diseases [37,38]. Although few studies have been conducted on PI3K/AKT in endometriosis [39], in one of the previous studies, phosphorylated AKT was observed in postmenopausal women with ovarian endometriosis [40], and phosphorylated mTOR was increased in ectopic lesions [41]. In the same context as prior studies, the results of this study indicated PI3K/AKT protein downregulation by 6,8-diprenylorobol with or without LY294002 in human endometriosis-like cell lines. In addition, temsirolimus, an mTOR drug that has exhibited the potential to reduce the size of endometrial lesions in vitro and in vivo, is currently approved for the treatment of renal cell carcinoma [42]. These results powerfully supported that the inhibition of the PI3K/AKT cell signaling pathway with 6,8-diprenylorobol can be a potential target in the treatment of endometriosis.

Our study included only cellular experiments without any animal or clinical trials yet. However, our in-depth study of the molecular mechanisms regulating the anti-cell-growth effects of 6,8-diprenylorobol on endometriosis could be a cornerstone for further studies. With further verification, 6,8-diprenylorobol might be used as a therapeutic agent in endometriosis progression.

## 5. Conclusions

The 6,8-diprenylorobol inhibited cell proliferation, with cell cycle arrest and calcium dysregulation via IP3 signaling. In addition, the PI3K/AKT proliferative cell signaling pathway was effectively decreased by 6,8-diprenylorobol. In addition, the increased P38 protein levels suppressed the cell viability of endometriosis-like cells. Moreover, the malfunction of the mitochondria, including loss of MMP, cellular respiration, and energy production, was mediated by 6,8-diprenylorobol treatment in the VK2/E6E7 and End1/E6E7 cell lines. All these results indicated the therapeutic potential of 6,8-diprenylorobol in human endometriosis.

## Data Availability

Data are contained within the article.
